# An unusual cause of female secondary infertility: Hypospadias

**DOI:** 10.4274/tjod.galenos.2020.30049

**Published:** 2020-10-02

**Authors:** Niyazi Tug, Mehmet Akif Sargin, Murat Yassa, Güldeniz Toklucu

**Affiliations:** 1 University of Health Sciences Turkey, Şehit Prof. Dr. İlhan Varank Sancaktepe Training and Research Hospital, Clinic of Obstetrics and Gynecology, İstanbul, Turkey

**Keywords:** Female hypospadias, female infertility, urethroplasty, sexual dysfunction, urinary tract infection

## Abstract

Female hypospadias is a very rare congenital anomaly and its impact on fertility has not yet been clearly defined. A 21-year-old woman with hypospadias was admitted with secondary infertility, dyspareunia, and urge symptoms. She was successfully treated with vaginal flap urethroplasty and broad spectrum antibiotics. Postoperatively, her symptoms resolved and she conceived spontaneously and aborted at her 17^th^ gestational week following premature rupture of membranes suggesting infection. She then conceived spontaneously again and delivered a healthy term baby 30 months after the operation. Female hypospadias may cause chronic pelvic infections, urge symptoms, sexual dysfunction, hence infertility with time. After achieving normal anatomy by vaginal flap urethroplasty, treatment of chronic infections allows restoring normal urologic and sexual functions, and fertility.

## Introduction

Female hypospadias, in particular among adults with no other genitourinary abnormalities, is a very rare clinical entity, with the urethral meatus located at any site on the anterior vaginal wall from just above the introitus up to the vaginal fornix^(1)^.

In hypospadias, urination into the vagina adversely affects the normal vaginal flora and favors cervico-vaginal infections, which may even lead to chronic or intermittent endometritis and infertility. Conversely, washing the vaginal secretions out of the vagina by urine would also cause vaginal dryness and hence dyspareunia which further contributes to the risk of infertility^(2)^.

In this paper, we report a 21-year-old woman with secondary infertility who presented with symptoms of dyspareunia, recurrent urinary tract infections, and urge symptoms. Her physical examination revealed hypospadias, and she was successfully treated with vaginal flap urethroplasty and delivered a healthy term baby.

## Case Report

**Figure 1:**
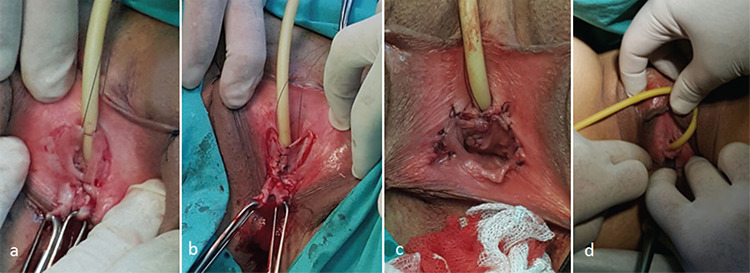
Urethroplasty operation. a) Perineal mucosa on both sides of the catheter, b) The incised mucosa was mobilized and sutured over the catheter, c) The vaginal strip was positioned and fixed over the neo-urethra, d) Two months postoperatively

A 21-year-old woman was admitted with severe dyspareunia, vaginal dryness, pelvic pain, and recurrent urinary tract infections. She had a healthy 6-year-old child as a result of sexual abuse but she did not describe any perineal trauma related with that. The patient was continent but reported severe urge symptoms. Her mother had worked as an agricultural laborer. Cervical cytology, vaginal culture and pelvic organs on transvaginal ultrasonography and hysterosalpingography were normal. The husband did not report any signs related to infertility and his spermiogram was normal. A physical examination revealed a urethral meatus located just above the hymenal ring and the vagina was prominently dry. On cystoscopy, chronic cystitis was diagnosed. Magnetic resonance imaging revealed grade 1 ureteropelvic ectasia. Female Sexual Function index (FSFI) was obtained from the patient^(3)^. Vaginal flap urethroplasty was performed. The surgical technique was as follows: A 16-French Foley catheter was inserted into the urethra. The anterior part of the hymenal ring and vaginal mucosa was incised with reverse U-shaped incision and dissected with its submucosa cephalad. The perineal mucosa anterior to the hymen was incised using railway shaped incisions on both sides of the catheter up to the point where normal urethral meatus was supposed to be located. This incised mucosa was mobilized and the flaps were sutured using separated sutures over the catheter to each other and to the original urethral meatus. The hymenal remnants of the other flap prepared from the hymenal ring and its continuing vaginal mucosa were extirpated. The prepared triangular vaginal flap edges were sutured at the midline to elongate the flap and a strip shape was obtained. The vaginal strip was then positioned over the neo-urethra and sutured to the neo-urethral meatus and to the intact perineal mucosa on both sides of the railway shaped incisions, tension-free. The 1-cm long spindle-shaped uncovered defects on each side of the neo-urethral meatus were closed using single sutures. For all sutures, 3/0 delayed absorbable material was used (Figure 1).

The patient was treated with broad spectrum antibiotics postoperatively. After two months of sexual abstinence, her initial symptoms were all resolved and her FSFI scores improved gradually at her 6^th^, 12^th^, and 24^th^ month follow-ups postoperatively. At her 14^th^ postoperative month, she conceived spontaneously but aborted at her 17^th^ gestational week following prematurely ruptured membranes and a bum-curettage was performed. She was then re-treated with broad spectrum antibiotics and she again conceived spontaneously. Then she delivered a term healthy 2900 g baby by elective cesarean section 16 months after the abortion.

## Discussion

The etiology of hypospadias is obscured in the majority cases. A multi-factorial explanation and the implication of genetic susceptibility and environmental pollutants remain a plausible working hypothesis^(4)^. In the presented case, the mother of the patient was an agriculture laborer, which suggests a possible *in utero *exposure to phytoestrogens or insecticides showing an anti-estrogenic effect as the etiologic factor.

Hypospadias results in urination into the vagina and hence, as in the presented case, causes recurrent urinary tract infections and disturbed normal vaginal flora, which play pivotal roles in the prevention of pelvic infections and infertility^(2)^.

Urine also washes the physiologic secretions out of the vagina and leads to vaginal dryness, which contributes to dyspareunia. In this case, the patient’s sexual functions gradually returned to normal after the operation as shown by the improvement in all items of the FSFI test in the 24-month follow-up (Table 1).

Female hypospadias is an unusual cause of infertility. In this case, the patient conceived her first pregnancy at age 14 years. Later, she conceived spontaneously after the operation but aborted following premature rupture of membranes. Following broad spectrum antibiotherapy she conceived again and delivered a healthy baby at term. This course of the patient’s medical data suggests an ascending pelvic infection secondary to impaired vaginal flora and cervicovaginitis as the cause of the infertility.

In this case, a female patient with hypospadias with urinary symptoms, sexual dysfunction, and infertility was treated successfully through urethroplasty surgery with vaginal flap.

## Figures and Tables

**Table 1:**
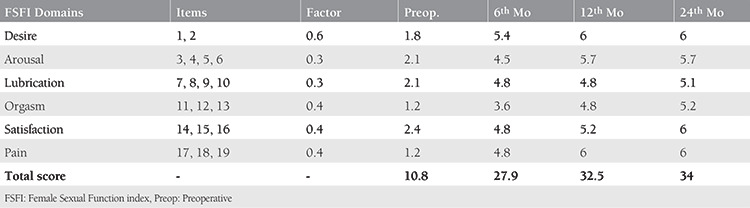
FSFI scores of the patient pre-operatively and 6, 12 and 24 months post-operatively. The gradual improvement of the scores is prominent
